# Relationship between nocturnal blood pressure dip and β-parapapillary atrophy zone choroidal vessel density in normal-tension glaucoma patients

**DOI:** 10.1371/journal.pone.0317468

**Published:** 2025-01-15

**Authors:** Jimin Park, Woo Keun Song, Min Su Baek, Jooyoung Yoon, Anna Lee, Ko Eun Kim, Michael S. Kook

**Affiliations:** Department of Ophthalmology, Asan Medical Center, University of Ulsan College of Medicine, Seoul, Korea; Tsukazaki Hospital, JAPAN

## Abstract

**Purpose:**

To investigate the relationship between nocturnal blood pressure (BP) dip and parapapillary choroidal vessel density (pCVD) in patients with normal-tension glaucoma (NTG)

**Methods:**

This study analyzed 267 eyes of 267 untreated NTG patients who underwent 24-hour (h) intraocular pressure (IOP) and ambulatory BP monitoring in the habitual position. Patients were classified into 3 groups [non-dippers (nocturnal BP dip < 10%), dippers (nocturnal BP dip between 10% and 20%, and over-dippers (nocturnal BP dip > 20%)], and pCVDs were measured by using optical coherence tomography angiography (OCTA) images. Logistic regression analyses were performed to identify clinical factors associated with “over-dipper” cases. Linear regression analyses were conducted to determine the correlation between various clinical variables and pCVD.

**Results:**

In clinical characteristics, over-dippers exhibited lower pCVD values compared to non-dippers or dippers (P *=* 0.004). High diurnal intraocular pressure (IOP) fluctuation (P *=* 0.031), high diurnal mean arterial pressure (MAP) fluctuation (P *=* 0.001), and low pCVD (P *=* 0.002) were identified as predictors of being “over-dipper” in multivariable logistic regression analyses. Moreover, peripapillary retinal vessel density (P *=* 0.040), presence of choroidal microvasculature dropout (P *=* 0.039), and nocturnal MAP dip % (P *=* 0.002) showed significant correlations with pCVD according to multivariable linear regression analyses.

**Conclusion:**

Over-dippers presented with lower pCVD than non-dippers or dippers as measured by OCTA choroidal images. Low pCVD was a predictor of “over-dipper” cases and associated with a greater percentage of nocturnal MAP dip in NTG patients. 24-h ambulatory BP monitoring may provide further information for detecting low pCVD in NTG patients with nocturnal BP dip.

## Introduction

The relationship between systemic hypotension and glaucoma has been thoroughly investigated in other clinical studies. Low systemic blood pressure (BP) has been identified as an important risk factor for the development and progression of open-angle glaucoma (OAG) [1–9]. Typically, a nocturnal BP dip, within the range of 10% to 20% relative to the diurnal BP, is physiologically observed in normotensive healthy subjects and most of hypertensive patients [10–12]. This nighttime BP reduction is referred to as a “dipper”, while patterns showing excessive dips (>20%) or minimal dips (<10%) during the night are referred to as “over-dipper” and “non-dipper”, respectively. An excessive nocturnal BP dip has been an indicative factor of vascular dysregulation [13–15] and has been linked to glaucoma development and progression in both normal-tension glaucoma (NTG) and OAG [4, 7, 16].

For the detection of systemic hypotension and nocturnal BP, continuous BP monitoring provides better information on both day and night BP changes than conventional snapshot diurnal BP readings [1, 7, 8]. An ambulatory blood pressure monitoring (ABPM) device provides 24-hour (h) BP data at high reproducibility [1, 2, 13]. Our recent studies have shown that extreme nocturnal hypotension, which causes significant fluctuation or variability in BP or mean ocular perfusion pressure (MOPP), is a consistent risk factor for NTG development and progression [17–20].

Optical coherence tomography angiography (OCTA) technology has opened new possibilities for non-invasively obtaining microvascular data, evaluating the perfusion status of the optic nerve head (ONH) and retinal layers with high repeatability. Several studies have now reported that a decreased parapapillary choroidal microvasculature may be related to the development and progression of glaucoma [21–24]. Other reports have further indicated that a focal loss in the parapapillary choroidal microvasculature, known as choroidal microvasculature dropout (CMvD), is associated with progressive changes in the retinal nerve fiber layer (RNFL) and with visual field (VF) damage in glaucoma patients [25–28]. Of interest in this regard, a recent study has demonstrated that CMvD is frequently observed in NTG patients with a nocturnal BP dip [29]. These findings highlight the importance of evaluating the parapapillary choroidal microvasculature using OCTA and its clinical relevance to glaucoma pathogenesis and management.

Considering that nocturnal hypotension indicates systemic vascular dysregulation and is associated with a microvasculature dropout at the parapapillary choroid in NTG patients [13–15, 29], we hypothesized that a nocturnal BP dip might be closely associated to a generalized insufficiency or loss of microvasculature in the parapapillary choroid among NTG patients. Hence, our current study evaluated the relationship between nocturnal hypotension and the parapapillary choroidal vessel density (pCVD) using OCTA technology in a large cohort of newly diagnosed NTG patients. We surmised that clarifying this relationship could offer significant insights into the role of a nocturnal BP dip in the ONH microvascular insufficiency, particularly in the parapapillary choroid, which is closely related to blood supply to deep ONH structures such as the prelaminar and laminar tissues [30, 31].

## Materials and methods

### Participants

This cross-sectional study included consecutive NTG patients from an ongoing prospective investigation evaluating the relationship between NTG and systemic BP. This prospective study began recruitment in January 2017 at Asan Medical Center Glaucoma Clinic, Seoul, Korea. The institutional review board of Asan Medical Center approved the present study, which was conducted in accordance with the principles of the Declaration of Helsinki. Every participant provided written informed consent.

Each participant underwent the following comprehensive ophthalmologic examinations: best-corrected visual acuity (BCVA) with manifest refraction, Goldmann applanation tonometry, slit-lamp biomicroscopy, gonioscopy, axial length (IOL Master; Carl Zeiss Meditec, Dublin, CA), central corneal thickness (CCT, DGH-550; DGH Technology, Exton, PA, USA), red-free fundus photography (AFC-210; Nidek, Aichi, Japan), stereoscopic optic disc photography, standard automated perimetry (Humphrey Field Analyzer with Swedish Interactive Threshold Algorithm (SITA)-Standard 24–2 VF test; Carl Zeiss Meditec, Dublin, CA, USA), Cirrus HD spectral domain optical coherence tomography (SD-OCT, Carl Zeiss Meditec, Dublin, CA, USA), and OCTA (Angiovue; Optovue Inc, Fremont, CA, USA).

The diagnosis of NTG was established by a glaucoma specialist (M.S.K.) with the following criteria: glaucomatous optic neuropathy (GON) with corresponding glaucomatous VF defects, intraocular pressure (IOP) less than 22 mmHg during outpatient clinic, and open anterior chamber angles on gonioscopy with no identifiable secondary cause of glaucoma. GON was defined as an asymmetric vertical cup-to-disc (C/D) ratio between the eyes greater than 0.2, a vertical C/D ratio over 0.7, unexplained by optic disc size, focal or generalized neuro-retinal rim thinning and/or RNFL defects [32]. CCT was measured three times at the initial visit using ultrasonic pachymetry, and an average value was documented. Glaucomatous VF defects was defined as the following criteria in two consecutive tests: 1) a cluster of 3 or more contiguous non-edge points on the pattern deviation probability plot with a probability less than 5% and with at least one of these points having a probability less than 1%; 2) glaucoma hemifield test results outside normal limits; or 3) pattern standard deviation (PSD) measured below 5% [33, 34]. The VF test was considered reliable on the bases of a false positive error <15%, false negative error <15%, and fixation loss <20%.

Newly diagnosed NTG patients in the outpatient clinic underwent 24-h IOP and ABPM monitoring upon admission. Patients were excluded from the study under the following criteria: outpatient IOP of more than 21 mmHg, a mean deviation (MD) worse than -12.00 dB at baseline in order to evaluate early-to-moderate stage NTG eyes to avoid confounding effect of advanced glaucoma severity on pCVD measurement [25], prior glaucoma or steroid treatments, a history of any intraocular or refractive surgery, any other ophthalmic or neurologic diseases that could affect the ONH evaluation or VF test, pathologic myopia, or lens opacity more than C2, N2, or P2 according to the Lens Opacities Classification System III that may affect proper VF testing [35]. Those who have a history of smoking, currently smoking, or having irregular sleeping patterns were also excluded. However, patients taking systemic antihypertensive agents were not excluded and were instructed to continue their use during the 24 h admission [20, 36, 37].

### Baseline in-hospital 24-h IOP and ABPM measurement

The method for obtaining an in-hospital 24-h IOP and ABPM has been described extensively in our prior studies [13, 17–20, 36, 37]. Briefly, IOP was measured by TonoPen XL (Mentor Ophthalmics, Santa Barbara, CA, USA). Diurnal IOP was measured every 2 hours (8 AM, 10 AM, 12 PM, 2 PM, 4 PM, 6PM, 8PM, and 10 PM) in a sitting position and nocturnal IOP was measured every 3 hours (12 PM, 3 AM, and 6 AM) in a supine position. The IOP was measured three times in each eye, and the average values were used in the analysis [20, 36, 37]. Various IOP parameters, including the mean, peak, trough, and fluctuations in the diurnal and nocturnal periods were calculated separately. Fluctuation was defined as the difference between the peak and trough IOP.

The systolic and diastolic BP levels, mean arterial pressure (MAP), and heart rate were monitored at 30-minute intervals over 24-h using a fully automatic ABPM device (Spacelabs Healthcare, Issaquah, WA, USA) [20, 36, 37]. This device was utilized to reduce examiner variability and measure BP in the most physiological setting. Patients were instructed to abstain from consuming alcohol and caffeine for three days prior to their admission. They were also advised to avoid physical activities that could affect the BP during their admission. The patients were instructed to remain indoors and continue their usual indoor activities during the daytime and sleep in a supine position using a flat pillow provided by the hospital with an 8-hour period of darkness during the night. Meals were provided at 7:30AM, 12:00PM, and 6:30PM.

### Definitions of nocturnal and diurnal BP dip

The MAP and the percentage of nocturnal and diurnal BP dip were calculated using the following equations, based on previous studies [20, 36, 37]. In this study, MAP was used as a surrogate BP parameter to represent both systolic and diastolic BP [10, 11]. The following equations were used:

MAP=DiastolicBP+[1/3×(SystolicBP-DiastolicBP)]

$$\text{NocturnalBP}\;\text{dip}\left(\%\right)=(\text{meandiurnal}\;\text{MAP}-\text{trough}\;\text{nocturnal}\;\text{MAP})/\text{mean}\;\text{diurnal}\;\text{MAP}\times 100$$

$$\text{DiurnalBP}\;\text{dip}\left(\%\right)=(\text{meandiurnalMAP}-\text{troughdiurnal}\;\text{MAP})/\text{mean}\;\text{diurnal}\;\text{MAP}\times 100.$$

Patients were categorized into three groups based on the percentage nocturnal MAP dip relative to the mean diurnal MAP as follows: non-dippers, less than 10% or negative dip (indicating a higher MAP during nighttime); dippers, 10% to 20%; over-dippers, more than 20% [10–12].

### Cirrus HD spectral-domain optical coherence tomography

By using Cirrus HD SD-OCT (software version 10.0), the circumpapillary RNFL thickness (cpRNFLT) and the macular ganglion cell-inner plexiform layer thickness (mGCIPLT) were measured. The average cpRNFLT was measured in a 3.45 mm circle by using the optic disc cube mode, scanning 6 × 6 mm^2^ ONH centered region, while the average mGCIPLT in the macular area within an annulus with outer vertical and horizontal diameters of 4 and 4.8 mm and inner vertical and horizontal diameters of 1 and 1.2 mm. Scans with good centration, signal strengths over 7, no movement artifacts, no poor clarity caused by artifacts and no segmentation errors were used in this study.

### Peripapillary retinal/macular and parapapillary choroidal vessel density measurement

All OCTA scans of the circumpapillary and macular area were assessed in this study using the AngioVue OCTA (software version 2018.1.1.69) to assure data consistency. This OCTA system provides a central wavelength of 840 nm, achieving a scanning speed of 70,000 A-scans per second. It offers an axial resolution of 5 μm and a transverse resolution of 15 μm. It employs a split-spectrum amplitude-decorrelation angiography algorithm to detect perfused vessels in various areas of the ONH and retina, which captures the dynamic movement of particles, such as red blood cells, to identify the blood flow [38].

For measuring macular vessel density (VD), all participants had macular imaging of a 6 × 6-mm^2^ scans centered on the fovea. An automated segmentation algorithm visualized the superficial retinal capillary plexuses, starting from the internal limiting membrane (ILM) to the posterior margin of the inner plexiform layer (IPL). Parafoveal and perifoveal macular VD (mVD) were analyzed by the AngioVue software. The parafoveal mVD and perifoveal mVD were measured within an annulus having inner and outer diameters of 1.0 mm and 3.0 mm and within an annulus having inner and outer diameters of 3.0 mm and 6.0 mm, respectively.

For measuring peripapillary retinal VD, all participants had peripapillary imaging of a 4.5 × 4.5 mm^2^ region with optic disc centering. The AngioVue software automatically measured the peripapillary retinal VD with a 1000-μm–wide elliptical annulus around the optic disc. The radial peripapillary capillary slab started from the internal limiting membrane to the nerve fiber layer, excluding large retinal vessels.

**Fig 1:**
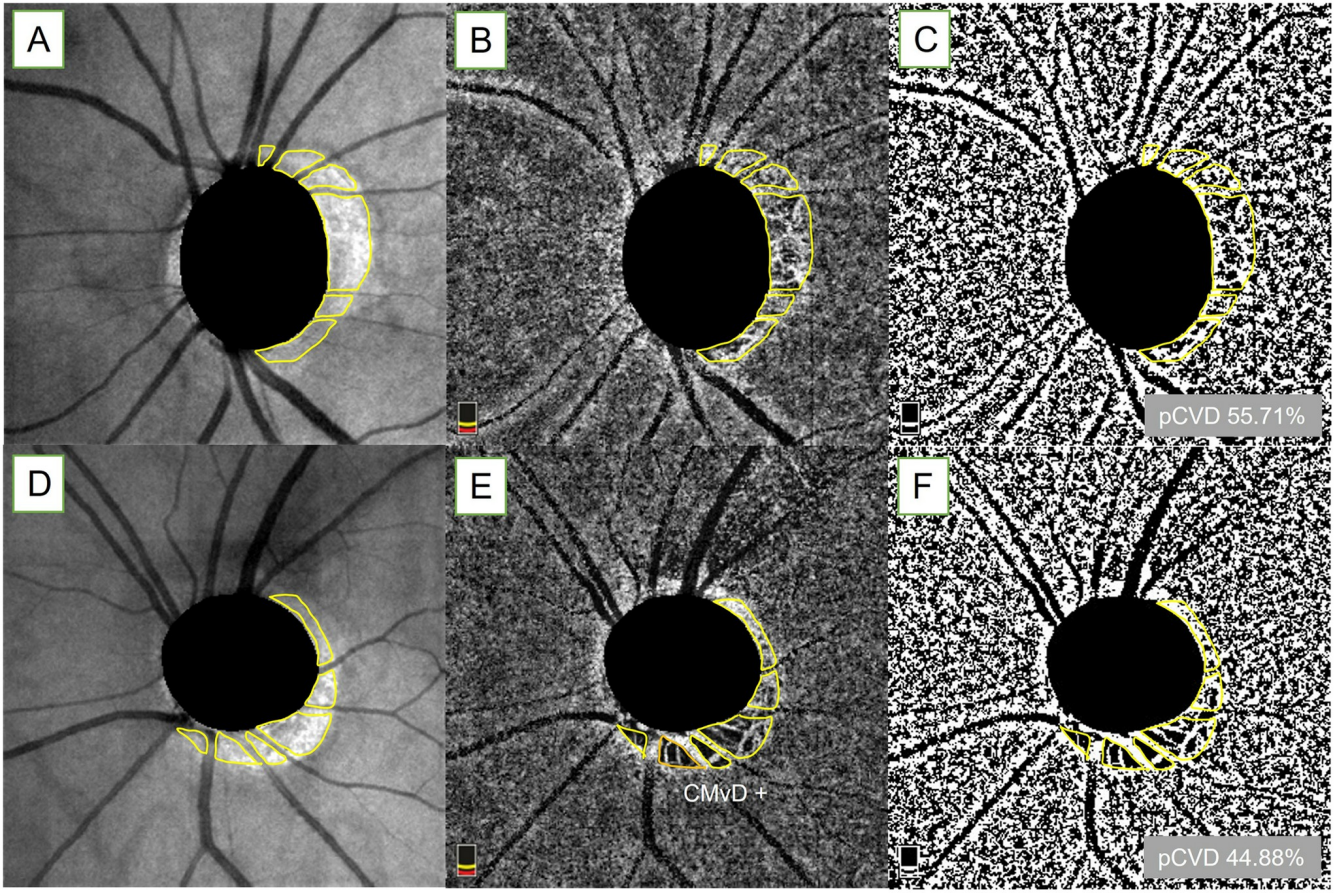
Measurement of parapapillary choroidal vessel density (pCVD). The boundaries of the optic disc and β-parapapillary atrophy margins, excluding large retinal vessels, were manually demarcated (yellow outline) using ImageJ software on en-face images obtained by using optical coherence tomography angiography scanning laser ophthalmoscopy (A and D). The demarcation lines are also shown above on en-face images of OCTA choroidal layer (B and E). The selected area of interest was converted to range of interest and 8-bit binary slab was created according to the mean threshold algorithm of ImageJ software of choroidal en-face image to measure pCVD (C and F). The orange demarcation outline shows choroidal microvascular dropout (CMvD) in figure E.

To measure the pCVD, method validated in previous studies was applied to OCTA choroidal images of a 4.5 × 4.5 mm^2^ region centering the optic disc [25–28, 39]. In brief, the margins of the optic disc and β-parapapillary atrophy (PPA) zone were manually outlined, while excluding the large overlying retinal vessels (Fig 1A, yellow line) on en-face images obtained by OCTA scanning laser ophthalmoscopy using ImageJ software (version 1.54; Wayne Rasband, National Institutes of Health, Bethesda, MD). The demarcation lines of the optic disc and β-PPA zone were also shown on en-face images of the parapapillary choroidal layer (Fig 1B, yellow line). By using the mean threshold algorithm of the imageJ software, an 8-bit binary slab was created on the choroidal layer image. The threshold value was set as the mean of the local grayscale distribution, which was produced automatically by the algorithm. After white pixels were assigned to vessels and black pixels to the background, the pCVD was calculated as a percentage of vessel pixels relative to the total number of pixels within the β-PPA area (Fig 1C, yellow outline). Two observers (J.P. and M.S.B.) who were blind to the clinical data independently measured the pCVD using the en-face images from the choroidal layer map of the OCTA. The averaged data of these two examiners were used in the final analyses. Another glaucoma case is presented to illustrate the pCVD measurement for comparison (Fig 1D–1F). The CMvD within the β-PPA was defined as a complete loss of the choriocapillaris and choroidal microvasculature, without any visible microvasculature network, in which the minimum width of the CMvD at the smallest part of the lesion was required to be 200 μm or greater than the width of the central retinal vein to avoid false positive findings as defined in previous studies (Fig 1E, orange outline) [24–29].

OCT-A images with the following criteria were excluded: (1) poor image quality due to low signal strength; (2) poor image clarity due to motion artifacts or media opacity; or (3) image with fixation error and segmentation failure [40]. Eyes without clear optic discs margins or β-PPA boundaries were also excluded.

### Statistical analysis

All statistical analyses were conducted using IBM SPSS Statistics 22 (SPSS Inc, Chicago, IL, USA). The inter-examiner agreement (J.P. and M.S.B) for the pCVD measurements was assessed using an intraclass correlation coefficient (ICC). The normality of the distribution was tested using a Kolmogorov-Smirnov test. Chi-square test was used to detect differences among the groups in categorical variables. One-way analysis of variance (ANOVA) was performed to detect differences among the non-dipper, dipper, and over-dipper groups with Bonferroni post-hoc test for multiple comparisons.

Univariable logistic regression analysis was used to determine potential clinical factors associated with “over-dipper” cases. A backward elimination process was conducted to build a multivariable logistic regression analysis incorporating variables with P values < 0.1 in univariable analysis. Since nocturnal MAP dip % was highly correlated with being an “over-dipper”, this factor was excluded as an independent variable in the logistic regression analysis to avoid collinearity. Finally, clinical factors correlated with the pCVD were assessed by linear regression analysis. Variables with P < 0.1 in the univariable analyses were counted as independent variables in the multivariable analysis. A backward elimination approach was used. P < 0.05 was considered significant in our multivariable analysis.

## Results

A total of 267 eyes from 267 NTG patients who met the inclusion criteria were analyzed. As indicated in Table 1, no significant differences were evident among the three patient groups in terms of demographic, ocular, and systemic features. There was an excellent inter-examiner agreement for the pCVD as measured by ICC between the two examiners (ICC = 0.9766). Among the 24-h IOP data, diurnal IOP fluctuation showed significant differences among the three groups, particularly between dippers and over-dippers. (P = 0.017, Bonferroni post hoc test: dippers vs over-dippers, P = 0.012). In the 24-h BP assessment, the diurnal MAP peak (P = 0.010), diurnal MAP fluctuation (P = 0.002), and diurnal MAP dip% (P = 0.032) showed significant differences among the three groups. The nocturnal MAP mean, peak, trough and fluctuation, and nocturnal MAP dip% also revealed significant differences among the three groups (all P<0.01, Table 2).

**Table 1:** Demographic and clinical characteristics of the subject eyes (patients) with normal-tension glaucoma in the non-dipper, dipper, and over-dipper groups.

Whole cohort (n = 267)	A. Non-dippers	B. Dippers	C. Over-dippers	p-value	A vs B	A vs C	B vs C
	(111 Eyes)	(97 Eyes)	(59 Eyes)		p-value	p-value	p-value
Demographic and ocular characteristics							
Age (year)	56.60±10.57	54.71±10.66	54.98±8.18	0.376	0.540	0.965	1.000
Male: Female	38:73	37:60	20:39	0.934	0.559	0.965	0.594
SE (diopter)	-1.92±3.03	-2.21±3.14	-2.43±3.11	0.568	1.000	0.925	1.000
CCT (μm)	539.65±32.57	538.97±33.56	537.25±30.57	0.902	1.000	1.000	1.000
AL (mm)	24.54±1.34	24.48±1.41	24.69±1.65	0.673	1.000	1.000	1.000
Outpatient IOP (mmHg)	14.40±2.00	14.13±2.18	14.14±1.68	0.575	1.000	1.000	1.000
VFI (%)	84.33±13.36	87.11±11.74	84.66±12.77	0.253	0.346	1.000	0.726
MD (dB)	-5.44±4.60	-4.49±4.10	-5.66±4.61	0.183	0.367	1.000	0.336
PSD (dB)	7.75±4.29	6.96±3.98	8.25±3.74	0.130	0.481	1.000	0.162
Disc hemorrhage (%)	13 (11.7%)	9 (9.3%)	8 (13.8%)	0.833	0.570	0.728	0.407
Systemic characteristics							
Family history (%)	13 (11.7)	13 (13.4)	7 (11.9)	0.929	0.714	0.977	0.781
Hypertension (%)	34 (30.6)	24 (24.7)	16 (27.1)	0.527	0.346	0.633	0.742
Diabetes mellitus (%)	8 (7.2)	4 (4.1)	2 (3.4)	0.246	0.343	0.315	0.818
CVA (%)	3 (2.7)	1 (1.0)	1 (1.7)	0.550	0.382	0.681	0.722
IHD (%)	2 (1.8)	1 (1.0)	0 (0.0)	0.289	0.643	0.301	0.435
Dyslipidemia (%)	15 (13.5)	19 (19.6)	6 (10.2)	0.789	0.238	0.529	0.121
Migraine (%)	4 (3.6)	3 (3.1)	3 (5.1)	0.684	0.839	0.645	0.532
Cold extremity (%)	10 (8.9)	6 (6.2)	2 (3.4)	0.157	0.447	0.175	0.444

SE, spherical equivalent; CCT, central corneal thickness; AL, axial length; Avg, average; CCT, central corneal thickness; VFI, visual field index; MD, mean deviation, PSD, pattern standard deviation; CVA, cerebrovascular accident; IHD, ischemic heart disease

*Significant P values (<0.05) are presented in bold.

**Table 2:** Comparison of in-hospital 24-hour IOP and BP data in the subject eyes (patients) with normal-tension glaucoma in the non-dipper, dipper, and over-dipper groups.

Whole cohort (n = 267)	A. Non-dippers	B. Dippers	C. Over-dippers	p-value	A vs B	A vs C	B vs C
	(111 Eyes)	(97 Eyes)	(59 Eyes)		p-value	p-value	p-value
IOP, mmHg							
Diurnal IOP mean	13.96±1.79	14.22±1.98	13.80±1.83	0.355	0.952	1.000	0.509
Diurnal IOP peak	16.33±2.04	16.34±2.27	16.29±2.24	0.988	1.000	1.000	1.000
Diurnal IOP trough	12.01±1.88	12.31±2.05	11.53±1.84	0.052	0.799	0.368	0.045
Diurnal IOP fluctuation	4.32±1.62	4.03±1.35	4.76±1.65	**0.017**	0.356	0.182	**0.012**
Nocturnal IOP mean	14.50±2.04	14.40±2.04	14.43±2.17	0.943	1.000	1.000	1.000
Nocturnal IOP peak	16.78±2.28	16.60±2.30	16.71±2.55	0.862	1.000	1.000	1.000
Nocturnal IOP trough	12.34±2.21	12.24±2.00	12.19±2.12	0.885	1.000	1.000	1.000
Nocturnal IOP fluctuation	4.43±1.91	4.36±1.67	4.53±2.05	0.866	1.000	1.000	1.000
MAP, mmHg							
Diurnal MAP mean	92.19±9.84	92.44±9.60	94.99±10.26	0.180	1.000	0.237	0.353
Diurnal MAP peak	103.45±11.42	103.97±11.16	108.93±12.55	**0.010**	0.945	**0.010**	0.027
Diurnal MAP trough	79.93±11.02	80.80±11.22	80.15±10.68	0.845	1.000	1.000	1.000
Diurnal MAP fluctuation	23.52±10.36	23.16±9.26	28.77±10.97	**0.002**	0.965	**0.004**	**0.003**
Nocturnal MAP mean	93.05±9.84	86.17±9.20	82.72±10.08	**<0.001**	**<0.001**	**<0.001**	0.079
Nocturnal MAP peak	98.25±11.41	93.43±10.49	94.63±12.53	**0.007**	**0.007**	0.120	0.797
Nocturnal MAP trough	88.22±9.22	78.88±8.41	69.40±9.03	**<0.001**	**<0.001**	**<0.001**	**<0.001**
Nocturnal MAP fluctuation	10.03±6.68	14.55±6.21	25.23±10.89	**<0.001**	**<0.001**	**<0.001**	**<0.001**
MAP dip %							
Diurnal MAP dip %	13.33±7.11	12.75±6.32	15.62±6.77	**0.032**	0.811	0.092	0.029
Nocturnal MAP dip %	4.18±4.37	14.65±2.74	26.90±5.50	**<0.001**	**<0.001**	**<0.001**	**<0.001**

IOP, intraocular pressure; MAP, mean arterial pressure.

*Significant P values (<0.05) are presented in bold.

Comparisons of OCT and OCTA parameters among the three groups are summarized in Table 3. While the groups did not significantly differ in terms of the average cpRNFLT, mGCIPLT, peripapillary VD, parafoveal VD, or perifoveal VD (all P > 0.05), the pCVD showed significant differences among the three groups (P = 0.004, Bonferroni post hoc test: non-dippers vs over-dippers, P = 0.003; Table 3).

**Table 3:** OCT/OCTA measurements of the subject eyes (patients) with normal-tension glaucoma in the non-dipper, dipper, and over-dipper groups.

Group (n = 267)	A. Non-dippers	B. Dippers	C. Over-dippers	p-value	A vs B	A vs C	B vs C
	(111 Eyes)	(97 Eyes)	(59 Eyes)		p-value	p-value	p-value
OCT thickness							
RNFLT (μm)	74.87±10.28	76.87±9.85	74.76±11.18	0.303	0.494	1.000	0.656
mGCIPLT (μm)	71.04±7.73	72.48±6.97	71.48±7.83	0.376	0.504	1.000	1.000
OCTA VD							
Peripapillary VD (%)	44.24±5.99	44.42±5.45	43.43±5.98	0.561	1.000	1.000	0.896
Parafoveal VD (%)	46.44±5.85	47.35±5.60	46.36±5.79	0.647	0.766	1.000	0.902
Perifoveal VD (%)	42.26±5.21	42.75±4.74	42.04±4.78	0.503	1.000	1.000	1.000
pCVD (%)	52.82±4.81	52.26±4.58	50.30±4.63	**0.004**	0.674	**0.003**	**0.031**

OCT, optical coherence tomography; RNFLT, retinal nerve fiber layer thickness; mGCIPLT, macular ganglion cell-inner plexiform layer thickness; OCTA, optical coherence tomography angiography; VD, vessel density; pCVD, parapapillary choroidal vessel density; n, number.

*Significant P values (<0.05) are presented in bold.

In the analysis of entire patients, diurnal IOP trough and fluctuation, diurnal MAP peak and fluctuation, pCVD, and diurnal MAP dip% were associated with being an “over-dipper” in univariable analyses (all P < 0.05 Table 4). By backward elimination, high diurnal IOP fluctuation (odds ratio (OR) 1.237; P = 0.031), high diurnal MAP fluctuation (OR 1.053; P = 0.031), and low pCVD (OR 0.905; P = 0.003) were significant predictors of being an “over-dipper” among these NTG patients in multivariable analysis (Table 4).

**Table 4:** Logistic regression analysis to determine the clinical factors related to “over-dipper”.

Variable	Univariate analysis			Multivariate analysis		
	OR	95% CI	P	OR	95% CI	P
Age	0.993	0.965–1.022	0.621			
Gender	1.100	0.598–2.021	0.760			
SE	0.962	0.877–1.056	0.415			
Family history	0.942	0.387–2.294	0.896			
Hypertension	0.962	0.503–1.842	0.908			
Diabetes mellitus	0.573	0.125–2.635	0.475			
CVA	0.879	0.096–8.021	0.909			
IHD	0	0	0.999			
Dyslipidemia	0.579	0.231–1.455	0.245			
Migraine	1.538	0.385–6.142	0.542			
Cold extremity	0.421	0.094–1.886	0.258			
CCT	0.998	0.989–1.007	0.666			
AL	1.092	0.886–1.342	0.402			
VFI	0.994	0.972–1.017	0.604			
MD	0.968	0.909–1.031	0.316			
PSD	1.053	0.982–1.130	0.148			
Diurnal IOP mean	0.920	0.786–1.077	0.299			
Diurnal IOP peak	0.990	0.865–1.132	0.879			
Diurnal IOP trough	0.844	0.722–0.985	**0.032**	Dropped		
**Diurnal IOP fluctuation**	1.259	1.049–1.511	**0.013**	1.237	1.019–1.502	**0.031**
Nocturnal IOP mean	0.995	0.864–1.145	0.940			
Nocturnal IOP peak	1.004	0.887–1.136	0.955			
Nocturnal IOP trough	0.976	0.850–1.120	0.731			
Nocturnal IOP fluctuation	1.037	0.889–1.209	0.644			
Diurnal MAP mean	1.028	0.998–1.058	**0.067**	Dropped		
Diurnal MAP peak	1.038	1.013–1.064	**0.003**	Dropped		
Diurnal MAP trough	0.998	0.972–1.025	0.909			
**Diurnal MAP fluctuation**	1.049	1.021–1.078	**0.001**	1.053	1.024–1.084	**<0.001**
RNFLT	0.990	0.963–1.018	0.497			
mGCIPLT	0.996	0.958–1.035	0.822			
Peripapillary VD	0.974	0.927–1.023	0.292			
Parafoveal VD	0.985	0.937–1.035	0.556			
Perifoveal VD	0.982	0.926–1.041	0.538			
Disc hemorrhage	1.326	0.558–3.154	0.523			
**pCVD**	0.899	0.842–0.961	**0.002**	0.905	0.847–0.967	**0.003**
Diurnal MAP dip %	1.052	1.011–1.095	**0.013**	**Dropped**		

SE, spherical equivalent; CCT, central corneal thickness; AL, axial length; Avg, average; CCT, central corneal thickness; VFI, visual field index; MD, mean deviation, PSD, pattern standard deviation; CVA, cerebrovascular accident; IHD, ischemic heart disease; IOP, intraocular pressure; MAP, mean arterial pressure, RNFLT, retinal nerve fiber layer thickness; mGCIPLT, macular ganglion cell-inner plexiform layer thickness; VD, vessel density; pCVD, parapapillary choroidal vessel density; CI, confidence interval

*P values <0.1 in univariate analysis and p values <0.05 in multivariate analysis are presented in bold.

In the entire group, the pCVD was significantly correlated with nocturnal MAP trough, nocturnal MAP fluctuation, peripapillary retinal VD and nocturnal MAP dip% in univariable analysis (all P < 0.05, Table 5). To minimize potential multicollinearity between the peripapillary VD and CMvD, [25] multivariable analyses were performed using two separate models, each incorporating variables with P ≤ 0.10 from the univariable analyses. In both models, the pCVD was consistently correlated with nocturnal MAP dip % (model 1: β-coefficient = -0.087; P = 0.004; model 2: β-coefficient = -0.090; P = 0.003) by backward elimination. In model 1, the pCVD was positively correlated with peripapillary VD (β-coefficient = 0.102; P = 0.040), while it was negatively correlated with presence of CMvD (β-coefficient = -1.219; P = 0.039) in model 2 (Table 5).

**Table 5:** Linear regression analysis for determining the clinical factors associated with the amount of pCVD.

	Univariate analysis			Multivariate analysis 1			Multivariate analysis 2		
Variable	β-Coefficient	95% CI	*P*	β-Coefficient	95% CI	*P*	β-Coefficient	95% CI	*P*
Age	-0.022	-0.079 to 0.035	0.440						
Gender	0.782	-0.418 to 1.982	0.201						
SE	0.031	-0.157 to 0.218	0.749						
Family history	-1.342	-3.085 to 0.402	0.131						
HTN	-0.979	-2.260 to 0.303	0.134						
Diabetes mellitus	-1.524	-4.103 to 1.054	0.245						
Dyslipidemia	-1.203	-2.811 to 0.405	0.142						
CVA	-2.628	-6.867 to 1.611	0.223						
IHD	0.889	-4.577 to 6.355	0.749						
Migraine	0.645	-2.389 to 3.679	0.676						
Cold extremity	-0.685	-2.982 to 1.612	0.558						
CCT	-0.008	-0.026 to 0.010	0.371						
AXL	-0.139	-0.552 to 0.274	0.507						
VFI	0.027	-0.018 to 0.073	0.236						
MD	0.060	-0.070 to 0.189	0.365						
PSD	-0.083	-0.224 to 0.058	0.250						
Diurnal IOP mean	0.079	-0.230 to 0.388	0.616						
Diurnal IOP peak	-0.046	-0.313 to 0.221	0.732						
Diurnal IOP trough	0.136	-0.159 to 0.431	0.365						
Diurnal IOP fluctuation	-0.304	-0.673 to 0.065	0.106						
Nocturnal IOP mean	0.016	-0.264 to 0.296	0.911						
Nocturnal IOP peak	0.009	-0.237 to 0.256	0.941						
Nocturnal IOP trough	0.062	-0.212 to 0.336	0.656						
Nocturnal IOP fluctuation	-0.065	-0.376 to 0.246	0.680						
Diurnal MAP mean	-0.001	-0.059 to 0.058	0.985						
Diurnal MAP peak	-0.008	-0.057 to 0.042	0.764						
Diurnal MAP trough	-0.025	-0.077 to 0.028	0.357						
Diurnal MAP fluctuation	0.018	-0.038 to 0.074	0.524						
Nocturnal MAP mean	0.037	-0.018 to 0.092	0.182						
Nocturnal MAP peak	0.004	-0.046 to 0.054	0.870						
Nocturnal MAP trough	**0.059**	**0.009 to 0.108**	**0.021**	Dropped			Dropped		
Nocturnal MAP fluctuation	**-0.078**	**-0.138 to -0.019**	**0.010**	Dropped			Dropped		
RNFLT average	0.007	-0.048 to 0.063	0.795						
mGCIPLT average	0.050	-0.027 to 0.127	0.202						
**Peripapillary VD average**	**0.109**	**0.010 to 0.208**	**0.031**	**0.102**	**0.004 to 0.200**	**0.040**			
Parafoveal VD average	**0.087**	**-0.013 to 0.187**	**0.089**	Dropped					
Perifoveal VD average	**0.113**	**-0.003 to 0.229**	**0.057**	Dropped					
**CMvD presence**	**-1.222**	**-2.397 to -0.048**	**0.041**				**-1.219**	**-2.375 to -0.062**	**0.039**
Disc hemorrhage	0.270	-1.555 to 2.094	0.771						
**Nocturnal MAP dip %**	**-0.900**	**-0.149 to -0.032**	**0.003**	**-0.087**	**-0.146 to -0.029**	**0.004**	**-0.090**	**-0.149 to -0.032**	**0.003**
Diurnal MAP dip %	0.070	-0.014 to 0.154	0.104						

pCVD, parapapillary choroidal vessel density; SE, spherical equivalent; CCT, central corneal thickness; AL, axial length; Avg, average; CCT, central corneal thickness; VFI, visual field index; MD, mean deviation, PSD, pattern standard deviation; CVA, cerebrovascular accident; IHD, ischemic heart disease; IOP, intraocular pressure; MAP, mean arterial pressure, RNFLT, retinal nerve fiber layer thickness; mGCIPLT, macular ganglion cell-inner plexiform layer thickness; VD, vessel density; CMvD, choroidal microvascular dropout

*P values <0.1 in univariate analysis and p values <0.05 in multivariate analysis are presented in bold.

**Fig 2:**
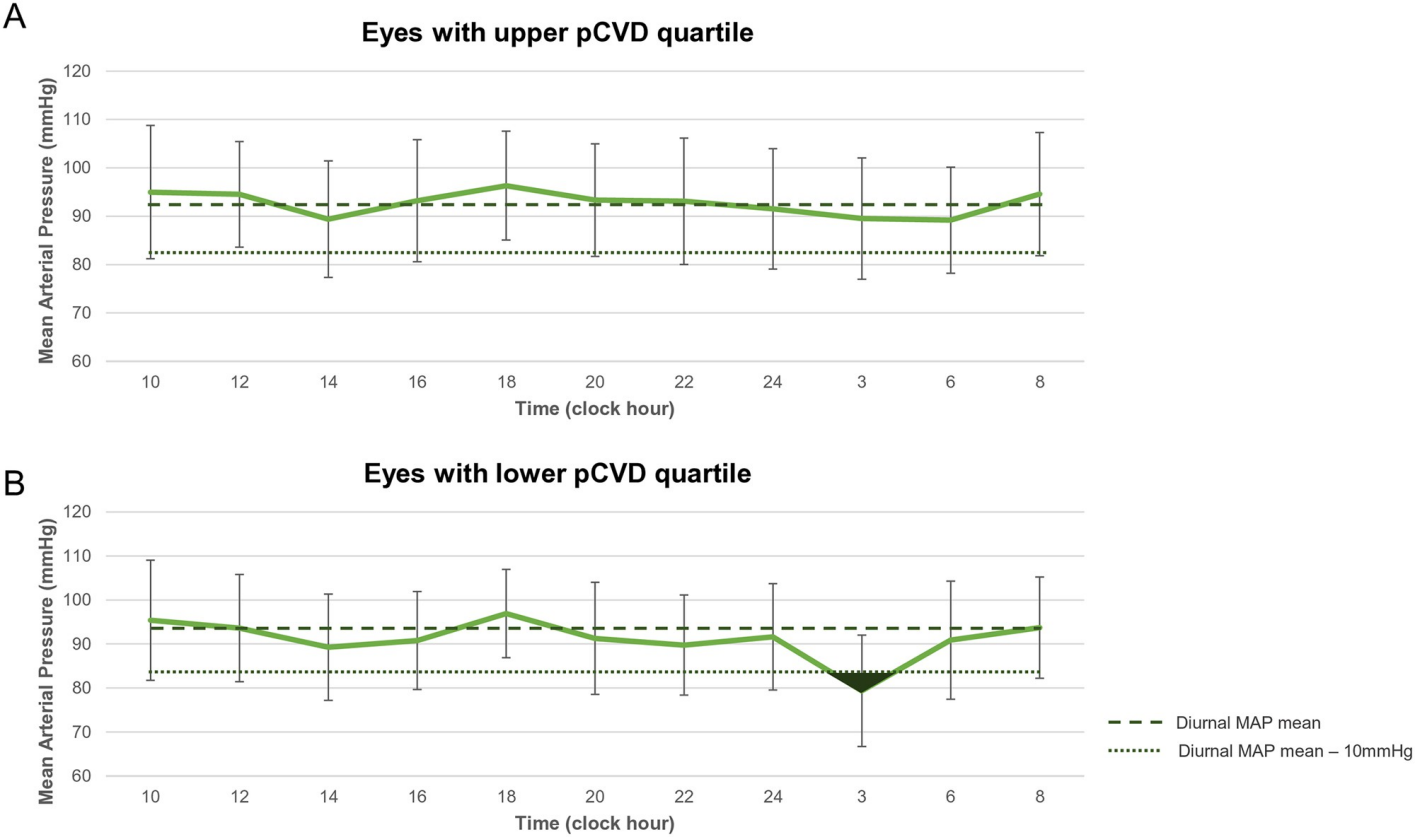
24-hour mean arterial pressure (MAP) patterns of upper and lower parapapillary choroidal vessel density (pCVD) quartile groups. The 2 dashed horizontal lines indicate diurnal MAP mean (upper panel) and diurnal MAP mean minus 10 mmHg (lower panel). Dark area represents nocturnal dip below diurnal MAP mean minus 10 mmHg.

As recent studies have revealed that a nocturnal BP lower than the diurnal MAP mean—10 mmHg may predict future NTG progression [7, 37], a 24-h MAP pattern of eyes in the upper and lower pCVD quartiles from our study cohort was drawn (Fig 2). While eyes in the upper pCVD quartile showed higher nocturnal MAP than the diurnal MAP mean—10mmHg throughout 24-h BP monitoring, those in the lower pCVD quartile demonstrated a lower MAP than diurnal MAP mean—10mmHg at around 3:00 AM, suggesting that a lower pCVD is associated with nocturnal BP dip in some NTG eyes.

**Fig 3:**
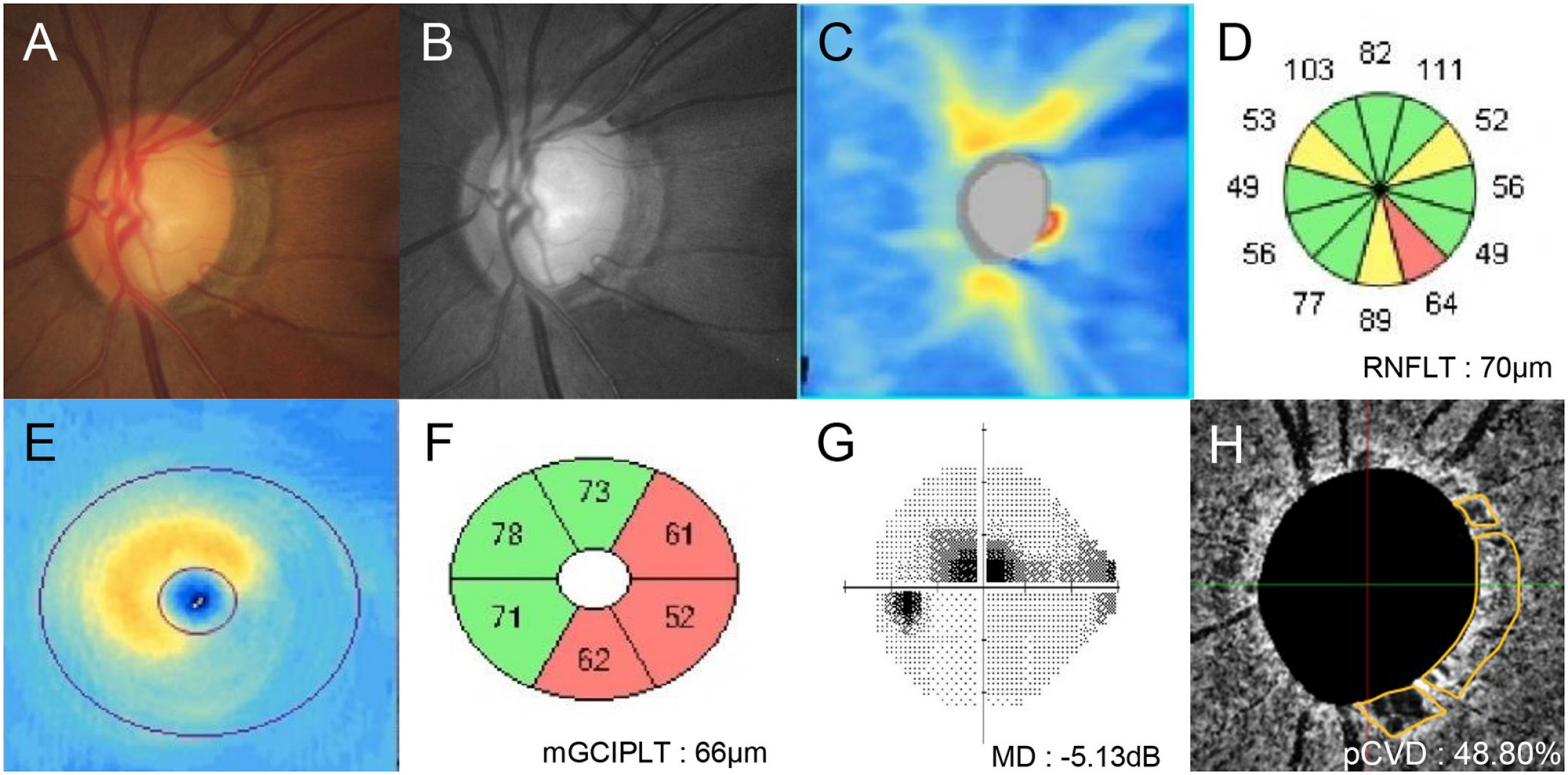
A representative case showing the relationship between parapapillary choroidal vessel density and nocturnal blood pressure dip. A 55-year-old over-dipper normal-tension glaucoma patient with axial length 23.49 mm and nocturnal blood pressure dip of 23.90% showed superotemporal and inferotemporal neural rim loss on optic disc photography and retinal nerve fiber layer (RNFL) loss on red-free RNFL photography. According to spectral-domain optical coherence tomography, RNFL and macular ganglion cell-inner plexiform thickness loss corresponding to the same locations of neural rim loss on optic disc photography were also evident. Visual field exam revealed corresponding central scotoma with a mean deviation of -5.13 dB. Parapapillary choroidal vessel density measurement using choroidal layer en-face images showed vessel density of 48.80%.

**Fig 4:**
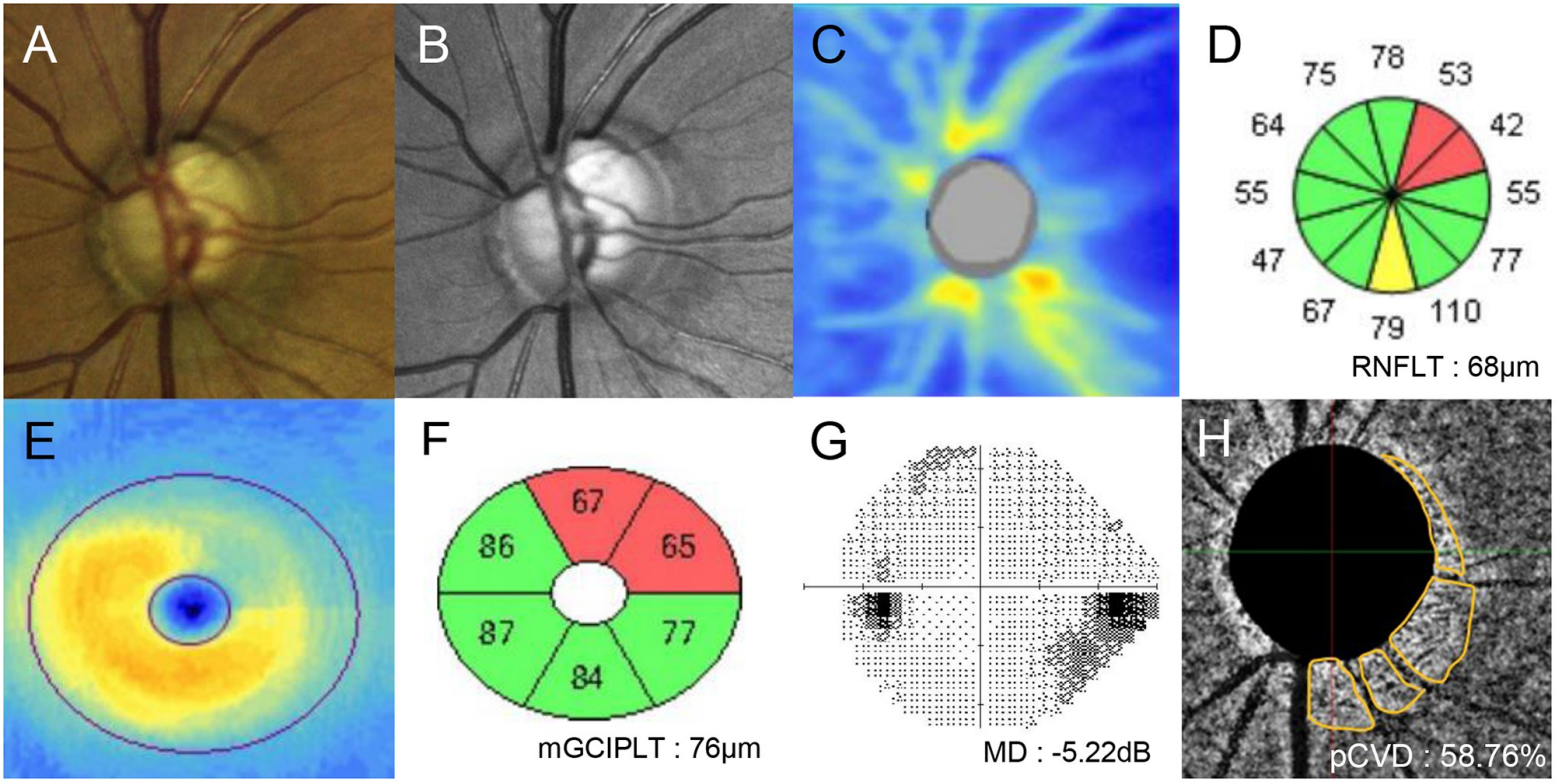
A representative case showing the relationship between parapapillary choroidal vessel density and nocturnal blood pressure dip. A 61-year-old non-dipper normal-tension glaucoma patient with axial length 23.54 mm and nocturnal blood pressure dip of 7.21% showed superotemporal neural rim loss on optic disc photography and retinal nerve fiber layer (RNFL) loss on red-free RNFL photography. Upon spectral-domain optical coherence tomography imaging, RNFL and macular ganglion cell-inner plexiform thickness loss corresponding with superotemporal location were also noted. Visual field exam showed corresponding inferonasal scotoma with a mean deviation of -5.22 dB. Parapapillary choroidal vessel density measurement by choroidal layer en-face images showed vessel density of 58.76%.

Representative cases from our current study cohort are shown in Figs 3 and 4. A 55-year-old over-dipper NTG patient with nocturnal BP dip of 23.90% and AL of 23.49 mm in the left eye showed superotemporal and inferotemporal neural rim loss on optic disc photography (Fig 3A), and RNFL loss on red-free RNFL photography in the same location (Fig 3B). On Cirrus HD SD-OCT, cpRNFLT reduction (Fig 3C and 3D) and mGCIPLT loss (Fig 3E and 3F) corresponding to superotemporal and inferotemporal locations and central VF scotoma were also noted (Fig 3G). This patient showed the pCVD of 48.80% (Fig 3H). In contrast, a 61-year-old non-dipper NTG patient with nocturnal BP dip of 7.21% and AL of 23.54 mm in the left eye showed superotemporal neural rim loss on optic disc photography (Fig 4A), and RNFL loss on red-free RNFL photography in the superotemporal location (Fig 4B). According to the Cirrus HD SD-OCT, cpRNFLT reduction (Fig 4C and 4D) and mGCIPLT loss (Fig 4E and 4F) corresponding to superotemporal location and inferonasal VF defects are also shown (Fig 4G). This patient showed pCVD of 58.76% (Fig 4H), which was relatively higher than that of over-dipper patient in Fig 3, despite having similar degree of cpRNFLT/mGCIPLT loss and VF severity between the two NTG patients.

## Discussion

We have here observed significant differences in the pCVD among three groups of NTG cases, in which over-dippers showed a significantly lower pCVD than non-dippers. Moreover, the pCVD was a significant predictor of being an “over-dipper” and was found to be closely related to the amount (%) of nocturnal MAP dip. While nocturnal hypotension is associated with ONH ischemia and is a significant risk factor for progressive VF loss in NTG eyes [7, 16, 37], no previous study has, to our knowledge, assessed the association between nocturnal BP dip and pCVD in NTG eyes using an OCTA technology and 24-h ABPM data. Our present findings may therefore provide valuable insights into the pathogenesis and clinical implications of pCVD in NTG patients.

In this study, 59 (22.1%) cases were classified as over-dippers based on our definition of a nocturnal BP dip of more than 20% relative to the mean daytime MAP [20, 36, 37], out of 267 newly diagnosed untreated NTG patients who were consecutively enrolled. This observation was in line with that of other previous studies which have reported a 10–30% prevalence of over-dippers among glaucomatous patients [4, 8, 36, 37]. In terms of demographic and clinical characteristics, we here found no significant differences among our three study groups, including systemic parameters (e.g., HTN, DM, etc.).

There were significant differences evident among our three groups with respect to 24-h IOP and BP parameters, as indicated in Table 2. These include diurnal IOP fluctuation, diurnal MAP parameters (peak and fluctuation), nocturnal MAP parameters (mean, peak, trough and fluctuation) and MAP dip% (diurnal and nocturnal), consistent with findings from recent studies [29, 37]. Our observations and previously reported results may collectively indicate that there is a positive relationship between the increased variabilities of 24-h IOP/BP parameters and being an “over-dipper” among NTG patients. Previous studies have revealed that variations in systemic and ocular hemodynamic parameters, such as the IOP and BP, may increase the vulnerability of the target organ, including ONH, via ischemia-reperfusion damage mechanisms [14, 19]. As over-dippers showed a greater degree of IOP and BP fluctuations compared to the other groups, including diurnal IOP fluctuation and diurnal/nocturnal MAP fluctuation (Table 2), these characteristics may be contributing risk factors for glaucoma progression among NTG patients who are over-dippers [20].

It was noteworthy there were no significant differences in OCT and OCTA parameters among our three groups except for the pCVD, as shown in Table 3. Based on our OCTA measurements, over-dipper group showed lower pCVD than either dippers or non-dippers, particularly compared to non-dippers (Table 3). Moreover, pCVD was a significant predictor of an “over-dipper” and showed a significant correlation with the amount (%) of nocturnal MAP dip in multivariable regression analyses. These findings may indicate a greater likelihood of global microvasculature insufficiency in the parapapillary choroid in over-dipper NTG cases.

Although it remains to be clarified, a possible explanation for the link between pCVD and a nocturnal BP dip is that a drop in BP at night could reduce perfusion in the posterior ciliary arteries (PCAs), which supply both the parapapillary choroid and the deep optic nerve tissues [30, 31]. Several end-organ tissues in the eye are auto-regulated in order to maintain constant microvascular perfusion. However, unlike other tissues such as the retina, the choroidal vascular circulation is not auto-regulated and mainly controlled by sympathetic innervation [41, 42]. Since sympathetic nerve activity gradually decreases at nighttime, while BP and its variability are lowered [10–12], excessive BP dips occurring on a repeated basis every night, as evident in our over-dipper patients, may lead to chronic perfusion deficiency in the PCAs, contributing to a significant VD reduction in the parapapillary choroidal region.

Our recent study demonstrated a significant association between CMvD within β-PPA and reduced pCVD in eyes with NTG [29]. CMvD may represent localized choroidal vascular impairment within the β-PPA near the ONH [24, 25], but it could also reflect generalized vascular insufficiency throughout the β-PPA. This interpretation is supported by the interconnected microvascular network of the parapapillary choroidal vessels within the β-PPA, which are supplied by the short posterior ciliary (SPC) artery [39]. Thus, CMvD might serve as a marker for broader choroidal vascular insufficiency in the β-PPA. Supporting this hypothesis, our study found that the presence of CMvD in the NTG cohort was significantly associated with reduced pCVD in the multivariable regression analysis (Table 5, P < 0.05).

In this study, we found a significant association between pCVD and peripapillary retinal VD based on multivariable regression analysis. However, no such association was observed between pCVD and RNFLT or mGCIPLT (Table 5). Previous studies have demonstrated a significant correlation between peripapillary retinal VD and other glaucoma parameters, such as RNFLT and VF MD [43, 44], as well as reductions peripapillary retinal VD in early OAG [45, 46]. Our findings indicate that pCVD may be closely associated with glaucoma severity, even though parameters like RNFLT, mGCIPLT, and VF indices did not show a similar association with pCVD in this study. While we cannot provide a definitive explanation for this observation, one possible hypothesis is that pCVD may reflect the vascular integrity of the ONH, with its reduction being more effectively captured by blood flow metrics like peripapillary retinal VD rather than other glaucoma measures such as RNFL or VF indices. Nonetheless, further studies are needed to replicate and validate these findings.

Recent studies have confirmed that the extent and duration of excessive nocturnal hypotension below the diurnal MAP mean-10 mmHg are significantly associated with glaucoma progression among NTG patients [7, 37]. Of interest in this regard, the lower pCVD quartile group in our present study demonstrated an average nocturnal MAP dip lower than the diurnal MAP mean-10 mmHg at around 3 AM, while our upper pCVD quartile group revealed an average nocturnal MAP that was consistently higher than the diurnal MAP mean-10 mmHg throughout the 24-h monitoring (Fig 3). Since a nocturnal MAP dip lower than the diurnal MAP mean-10 mmHg may indicate an excessive nocturnal BP drop beyond the range of the physiological BP at nighttime [7, 37], our findings may suggest that an excessive nocturnal MAP dip is closely linked to microcirculatory insufficiency to the parapapillary choroid as represented by a lower pCVD from compromised blood flow to the PCAs. Furthermore, this phenomenon may contribute to glaucomatous progression in some NTG patients [7, 37], since a decreased blood flow to the PCAs and ONH is an established risk factor for glaucoma progression [47, 48].

Although our current study is the first large-scale investigation of the impact of nocturnal BP dip on pCVD among NTG patients using 24-h ABPM and OCTA data, it had several limitations of note. First, no precise information regarding the amount and type of oral antihypertensive medications was available because patients self-reported the information. Even though hypertension was not found to be a significant risk factor for being an “over-dipper” in our present analyses, any use of oral antihypertensive medications might have had an independent influence on the relationship between nocturnal BP dip and pCVD. Further studies are needed to evaluate the impact of different types and amounts of oral antihypertensive agents on 24-h ABPM readings and relationship with pCVD in glaucoma patients. Another limitation is that the measurement of the pCVD can be affected by OCTA-associated noise and subjectivity. Measurement artifacts, such as shadow effects or projection of overlying retinal vessel signals, may lead to under- or over-estimation of this variable and can hinder its precise measurement [26–29]. Moreover, subjectivity in the process of delineating the ONH margin and β-PPA zone can introduce inaccuracy in pCVD measurements [26, 29]. To reduce the impact of the aforementioned limitations, only good-quality OCTA images were used, while applying strict exclusion criteria. Moreover, the pCVD was measured by two examiners using a method that has been validated in previous studies [23, 25–29], with an excellent inter-observer agreement between our two independent examiners (an ICC of 0.9766). Third, as our patients were enrolled from a tertiary referral center, this may have led to a patient selection bias, not representing general population. Fourth, our patients consisted of Korean NTG patients, our findings may not be fully applicable to other populations or to other types of glaucoma. Lastly, because of our cross-sectional study design, no longitudinal relationship was assessed between the nocturnal BP dip and loss of pCVD. Moreover, without longitudinal data, it is unknown whether lower pCVD associated with nocturnal BP dip increases the likelihood of future glaucoma progression. Prospective, longitudinal studies are thus needed to clarify not only the relationship between nocturnal BP dip and progressive pCVD reduction but also that between pCVD reduction related to nocturnal hypotension and future glaucoma progression.

## Conclusions

In conclusion, a lower pCVD is a significant predictor of an “over-dipper” NTG patient and is significantly correlated with a higher percentage of nocturnal MAP dip. While 24-h ABPM offers valuable information, including nocturnal BP dip and its role in the pathogenesis of glaucoma in NTG patients, it may pose a practical challenge in terms of its wider use among NTG patients. Since our present findings demonstrated that the NTG patients with a low pCVD are closely associated with a greater nocturnal BP dip, these patients may be selectively recommended in future to undergo 24-h ABPM to detect a possible nocturnal BP dip.

## Supporting information

S1 Data (XLSX)
